# Silica nanoparticles with encapsulated DNA (SPED) to trace the spread of pathogens in healthcare

**DOI:** 10.1186/s13756-021-01041-3

**Published:** 2022-01-10

**Authors:** Cinzia Ullrich, Anne M. Luescher, Julian Koch, Robert N. Grass, Hugo Sax

**Affiliations:** 1 Department of Infectious Diseases and Hospital Epidemiology, University Hospital Zurich, University of Zurich, Zurich, Switzerland; 2 Department of Chemistry and Applied Biosciences, Institute for Chemical and Bioengineering, ETH Zurich, Vladimir-Prelog-Weg 1-5/10, 8093 Zurich, Switzerland; 3 Department of Infectious Diseases, Bern University Hospital and University of Bern, Friedbuehlstrasse 53, 3010 Bern, Switzerland

**Keywords:** Infection control, Infection prevention, Pathogen transmission, Silica nanoparticles, Surrogate markers

## Abstract

**Background:**

To establish effective infection control protocols, understanding pathogen transmission pathways is essential. Non-infectious surrogate tracers may safely explore these pathways and challenge pre-existing assumptions. We used silica nanoparticles with encapsulated DNA (SPED) for the first time in a real-life hospital setting to investigate potential transmission routes of vancomycin-resistant enterococci in the context of a prolonged outbreak.

**Methods:**

The two study experiments took place in the 900-bed University Hospital Zurich, Switzerland. A three-run ‘Patient experiment’ investigated pathogen transmission via toilet seats in a two-patient room with shared bathroom. First, various predetermined body and fomite sites in a two-bed patient room were probed at baseline. Then, after the first patient was contaminated with SPED at the subgluteal region, both patients sequentially performed a toilet routine. All sites were consequently swabbed again for SPED contamination. Eight hours later, further spread was tested at predefined sites in the patient room and throughout the ward. A two-run ‘Mobile device experiment’ explored the potential transmission by mobile phones and stethoscopes in a quasi-realistic setting. All SPED contamination statuses and levels were determined by real-time qPCR.

**Results:**

Over all three runs, the ‘Patient experiment’ yielded SPED in 59 of 73 (80.8%) predefined body and environmental sites. Specifically, positivity rates were 100% on subgluteal skin, toilet seats, tap handles, and entertainment devices, the initially contaminated patients’ hands; 83.3% on patient phones and bed controls; 80% on intravenous pumps; 75% on toilet flush plates and door handles, and 0% on the initially not contaminated patients’ hands. SPED spread as far as doctor’s keyboards (66.6%), staff mobile phones (33.3%) and nurses’ keyboards (33.3%) after eight hours. The ‘Mobile device experiment’ resulted in 16 of 22 (72.7%) positive follow-up samples, and transmission to the second patient occurred in one of the two runs.

**Conclusions:**

For the first time SPED were used to investigate potential transmission pathways in a real hospital setting. The results suggest that, in the absence of targeted cleaning, toilet seats and mobile devices may result in widespread transmission of pathogens departing from one contaminated patient skin region.

## Background

Healthcare associated infections (HCAI) cause higher complication and mortality rates, prolonged hospital stays, and increased healthcare costs [1, 2]. Moreover, the spread of antimicrobial resistant pathogens is considered one of the most crucial issues in healthcare [3].

Multiple large-scale outbreaks of vancomycin-resistant enterococci (VRE) in Swiss hospitals in the last decade indicate a surge of VRE in Switzerland [4–6]. Poor hand hygiene (HH) has proven to be a primary cause of pathogen transmission [7]. Guidelines and protocols have been established to improve hand hygiene in healthcare [2, 8] with limited overall success [9, 10]. Several studies published over the past 20 years indicate the effect of contaminated hospital environment on pathogen transmission—especially multi-resistant pathogens such as VRE [11–14]. Pittet et al. [7] list “organisms shed onto inanimate objects immediately surrounding the patient” as the starting point of cross-transmission between patients. Other studies suggest that the risk for VRE acquisition is higher when a patient stays in a hospital room previously occupied by a VRE-infected patient [15–17]. Lower rates of multi-drug resistant pathogen colonization and infection occur in hospitals with single-rooms and thus, individualized toilet use [18, 19].

Touching a VRE-contaminated surface carries a similar risk for pathogen-transmission on hands as touching a colonized patient even though the concentration of VRE on surfaces is much lower [12, 20, 21]. Randle et al. [22] found that HH compliance in HCW was 80% [adjusted odds ratio (aOR) 1.88, 95% CI 1.15–3.07)] after direct patient contact, whereas HH was performed in only 50% (aOR 0.60, 95% CI 0.38–0.93) after contact with a patient’s surroundings. In an observational study with head cameras during real-life active patient care Clack et al. even found a HH “adherence” of only 5% prior to potentially patient colonizing touch events and only 1% before possible infection events (e.g., before touching central line insertion sites, wounds, sterile needles) [10].

It is not surprising that Cassone et al. [23] included toilet seats in an environmental panel as a proxy for patients with VRE colonization. Although many experts emphasize the transmission of pathogens from contaminated areas through the hands of healthcare workers (HCW), fewer recognize the effect of shared bathrooms or toilets as possible transmission hubs of multi-drug resistant microorganisms [24]. The transmission pathway of pathogens, in particular VRE, through toilet seats has not yet been explicitly investigated.

The use of surrogate tracers allows to safely determine patient-to-patient-transmission of pathogens in real-life care-settings [25, 26]. In the past, various pathogen surrogates have been used, including cauliflower mosaic virus DNA [27–31], bacteriophage MS-2 [29, 32, 33], non-toxigenic *Clostridioides difficile* spores [34], fluorescent agents [35], and light-reflecting chemical compounds combined with flashlight photography [33]. Silica nanoparticles with encapsulated DNA (SPED) with known nucleotide sequences have been described by Paunescu et al. [36] (Fig. 6). They are used as inert tracers in biological product tagging, tracing of food, studying animal predator–prey-relationships, and characterizing aquifer and wastewater [37–40]. As Scotoni et al. [25] have shown in a microbiology and a behaviour laboratory setting, that SPED represent promising surrogate tracers for microbial transmission in healthcare because SPED and bacteria share strong similarities in transmission, are non-toxic, and can be individually tagged [41].

In the current work, we aimed to investigate the spread of SPED as surrogates for pathogen transmission between patients in a real-world-scenario, involving the shared use of the toilet in a two-bed patient room. As SPED are insensitive to common disinfectants this study aims to display transmission pathways in an environment without disinfection procedures. This investigation was clinically motivated by an ongoing VRE outbreak in our hospital in the course of which the risk of shared restroom use and necessary cleaning schedules became a topic of interest.

## Methods

### SPED and swab technique

The synthesis and characterization of SPED followed the protocol of Paunescu et al. [36]. We used the same three batches of SPED with individual DNA-tagging as previously described by Scotoni et al. [25], SPED-1 (218 ± 80 nm; DNA loading 21 µg), SPED-2 (146.6 ± 46 nm; DNA loading 23 µg), and SPED-3 (173.4 ± 82 nm; DNA loading 26 µg). The nature and handling of SPED is described in detail in the Technical Appendix.

### Quantification of SPED and statistical analysis

The procurance of exact SPED concentration levels based on real-time quantitative PCR (qPCR) cycle values is specified in the Technical Appendix. Before deposition of SPED every swabbing site was swabbed as baseline sample. To evaluate the corresponding concentrations in mg/mL, all samples were compared with an experiment-specific threshold resulting from the SPED concentration of the baseline sample yielding the strongest qPCR signal and thus, the highest concentration of all baseline samples. This represents a conservative approach to adjust all results for background signals resulting from contamination arising during experimental processing. Consequently, values with concentrations above threshold were considered as positive, values below as negative. The descriptive analysis was conducted with Microsoft Excel® 2020.

### Explorative experiments

Before their use with patients, we chose to explore SPED for transmission characteristics in relation to the necessary amount and concentration in the restroom of an office building of the hospital campus in two controlled experiments.

First, 3 mL of a 1 mg/mL SPED suspension in ultrapure MilliQ water (type 1, 18.2 MΩ·cm at 24 °C, Milli-Q®; Merck, Darmstadt, Germany) were deposited on bare skin at a subgluteal skin site of a voluntary 27-year-old male participant and left to air-dry. The participant was then asked to sit on the toilet seat for ten seconds, flush the toilet, and perform handwashing after having left the restroom by using the door handle. The participant's contaminated body site, toilet seat, toilet flush plate, door handle, and tap were swabbed before and after this sequence. In a follow-up experiment, we verified the transmission of SPED from a Participant-A, contaminated equally as described above, to Participant-B, both subsequentially performing the same toilet use sequence as described above.

### Patient experiment

**Fig. 1:**
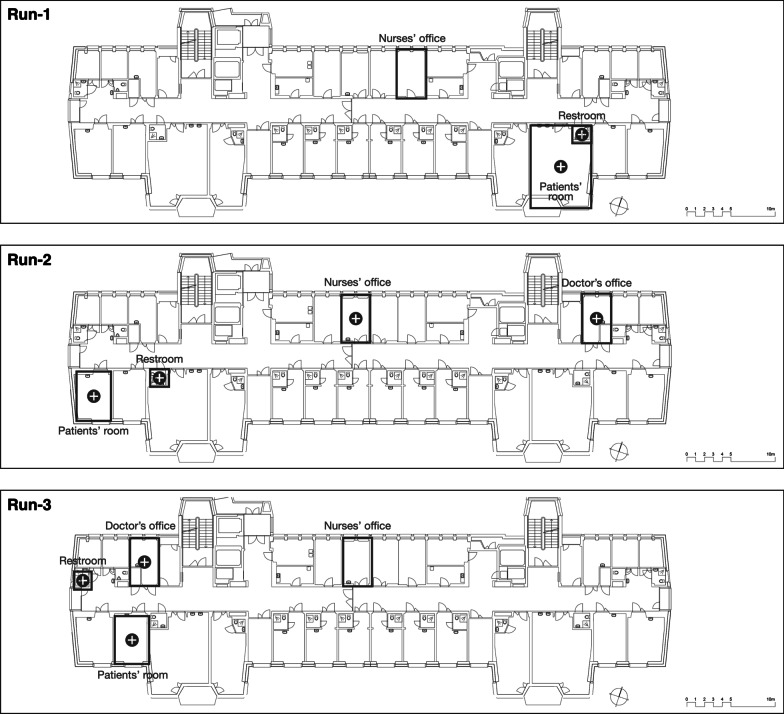
Floor plan of the ‘Patient experiment’ ward with room assignments. The floorplan shows the study ward three times (once for each study run) and the localisation of the two-bed patient room, shared restroom, nurses’ and doctor’s office in the study ward. In Run-1 the doctor’s office was situated on another floor and does therefore not appear in the floor plan. The plus signs indicate rooms with one or more positive SPED swabbing results at 8-h follow-up. Detailed swabbing sites s. Table 1. *SPED* silica nanoparticles with encapsulated DNA

The patient experiment took place in patient rooms on a haemato-oncological ward at the University Hospital Zurich, Switzerland, it was repeated three times (Run-1–Run-3) with a new SPED batch being used (SPED-1–SPED-3) for each run. Run-1 and Run-2 took place in the same, Run-3 in another patient room of similar layout (Fig. 1). During the experiment, cleaning in the patient room was limited to floor wiping.

Any pair of patients in a two-bed room were eligible for the study if they both gave informed consent. They were conveniently chosen by ward staff and allowed to withdraw at any time without indicating a reason. The ward staff was collectively informed about the study with the possibility to opt out.

We pre-defined 20 swabbing sites dimensioned 1 × 3 cm, 16 concerning fomites, of which eight were located inside, four in the restroom and four outside the patient room, and two body sites on each patient (Table 1). These swab sites were chosen following the idea of ‘high-touch surfaces’ according to Huslage et al. [42]. Swabbing was performed as described in the “Technical Appendix”.

**Table 1:** Swabbing sites in the ‘Patient experiment’ for all three runs

	Sampling sites before deployment of SPED at baseline	Sampling sites immediately after toilet sequence	Sampling sites after eight-hour interval
*Patient skin*			
Subgluteal skin patient A	X	X	
Hands/palms patient A	X		X
Subgluteal skin patient B	X	X	
Hands/palms patient B	X		X
*Restroom*			
Toilet seat after patient A	X	X	
Flush plate after patient A	X	X	
Tap handle after patient A	X	X	
Door handle after patient A	X	X	
Toilet seat after patient B	X	X	
Flush plate after patient B	X	X	
Tap handle after patient B	X	X	
Door handle after patient B	X	X	
*Patient room*			
Bed control patient A	X		X
Private phone patient A*	X		X
Entertainment device patient A†	X		X
Intravenous pump patient A	X		X
Bed control patient B	X		X
Private phone patient B*	X		X
Entertainment device patient B†	X		X
Intravenous pump patient B	X		X
*Nurse’s office*			
Nurse’s mobile phone	X		X
Nurse’s office keyboard	X		X
*Doctor’s office*			
Doctor’s mobile phone	X		X
Doctor’s office Keyboard	X		X

*SPED* silica nanoparticles with encapsulated DNA

*The patients’ private mobile phone was swapped if available, otherwise the beside phone provided by hospital

^†^The most used device was assessed (among tablet, e-reader, laptop, hospital bedside TV): Run 1 A: tablet, B: bedside hospital TV; Run 2 A: e-reader, B: bedside hospital TV; Run 3 A: tablet, B: bedside hospital TV

First, all sites were swabbed as negative controls. Then, we applied 3 mL of our SPED test suspension (1 mg/mL) to the subgluteal region of Patient-A by using a graduated pipette and a brush and left it to air-dry. A swab from the subgluteal swabbing sites of Patient-A taken immediately after contamination constituted the positive control. Consecutively, we asked Patient-A to dress, go to the bathroom and sit on the toilet seat for 10 s. Then Patient-A would dress, flush the toilet, perform handwashing with soap and water, open the bathroom door and go back to bed. While performing this toilet sequence Patient-A was not observed, leaving it therefore open whether Patient A touched the toilet ring, his/her own subgluteal region or no fomite at all. Immediately after this sequence, we took swabs of Patient-A’s subgluteal skin as well as from the toilet seat, tap handle, flush plate, and bathroom door handle. Subsequently, Patient-B was asked to perform the same toilet use sequence as Patient-A. We again took samples from the predefined Patient-B and environmental swabbing sites. Then, the researcher left the ward asking both patients to behave as usual.

After an eight-hour interval, the researcher returned to the ward and sampled both patients’ hands and three predefined environmental sites for each patient (i.e., bed control unit, phone, the personal entertainment device that the patient reported to have mainly used among laptop, e-reader, tablet, hospital-bedside TV, and intravenous pump). Keyboards and mobile phones of the nurse and the physician intern in charge of Patient-A and Patient-B were swabbed accordingly (Table 1). No decontamination process was applied after the SPED experiments.

### Mobile device experiment

To investigate whether SPED transmit from one patient to another through HCW mobile phones, we conceived an additional quasi-realistic experiment with the help of healthy volunteers.

**Fig. 2:**
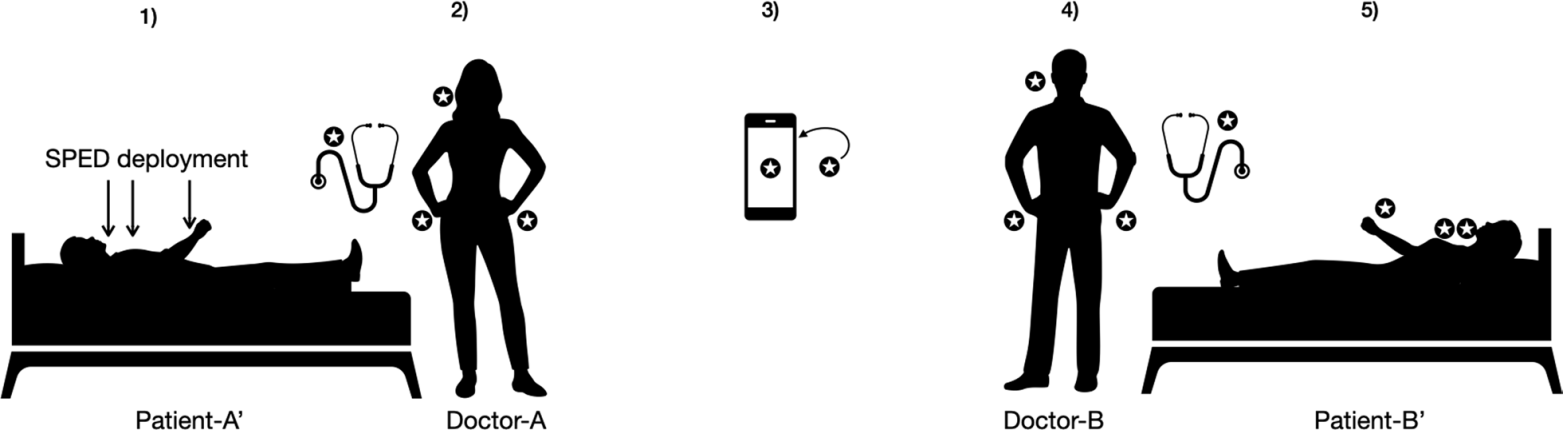
Setup of the ‘Mobile device experiment’. Scenarios plot: (1) Researcher deploys SPED on neck (A. carotis), chest (Erb’s point), and wrist (A. radialis) of Patient-A′; (2) Doctor-A listens to heart sounds and takes radial pulse of Patient-A′; (3) Phone rings, Doctor-A takes the call for 30 s, then leaves the experiment scene; (4) Phone rings again, Doctor-B takes the call for 30 s; and (5) listens to heart sounds and takes radial pulse of Patient-B′. Star symbols indicate SPED swabbing sites. *SPED* silica nanoparticles with encapsulated DNA.

Four volunteers performed a standardized scenario as Patient-A′, Patient-B′, Doctor-A, and Doctor-B in a simulated two-bed hospital room involving personal stethoscopes and a single shared mobile phone (Fig. 2), repeated as Run-1′ and Run-2′ using SPED-3 and SPED-2, respectively. SPED were deployed on Patient-A’s wrist, chest, and neck. Then, Doctor-A examined Patient-A′, answered the mobile phone, which was consequently used by Doctor-B, who in turn examined Patient-B′. Before SPED deployment and after, the experiment swabs were taken from the patients’ wrists, neck and chest, both doctors’ hands and cheeks, and both stethoscopes as well as the front and back of the mobile phone (Table 2).

**Table 2:** Swabbing sites in the ‘Mobile device experiment’ for both runs

	Swabbing sites before deployment of SPED as baseline	Swabbing sites after patient care sequence
*Patient A′*		
Wrist, radial pulse point	X	X
Neck, carotid pulse point	X	X
Chest, Erb’s point	X	X
*Doctor A*		
Cheek, phone touching point	X	X
Hands, palms	X	X
Stethoscope A, chest piece	X	X
*Patient B′*		
Wrist, radial pulse point	X	X
Neck, carotid pulse point	X	X
Chest, Erb’s point	X	X
*Doctor B*		
Cheek, phone touching point	X	X
Hands, palms	X	X
Stethoscope B, chest piece	X	X
*Mobile phone*		
Phone, frontside screen	X	X
Phone, backside	X	X

*SPED* silica nanoparticles with encapsulated DNA

## Results

### Explorative experiments

**Fig. 3:**
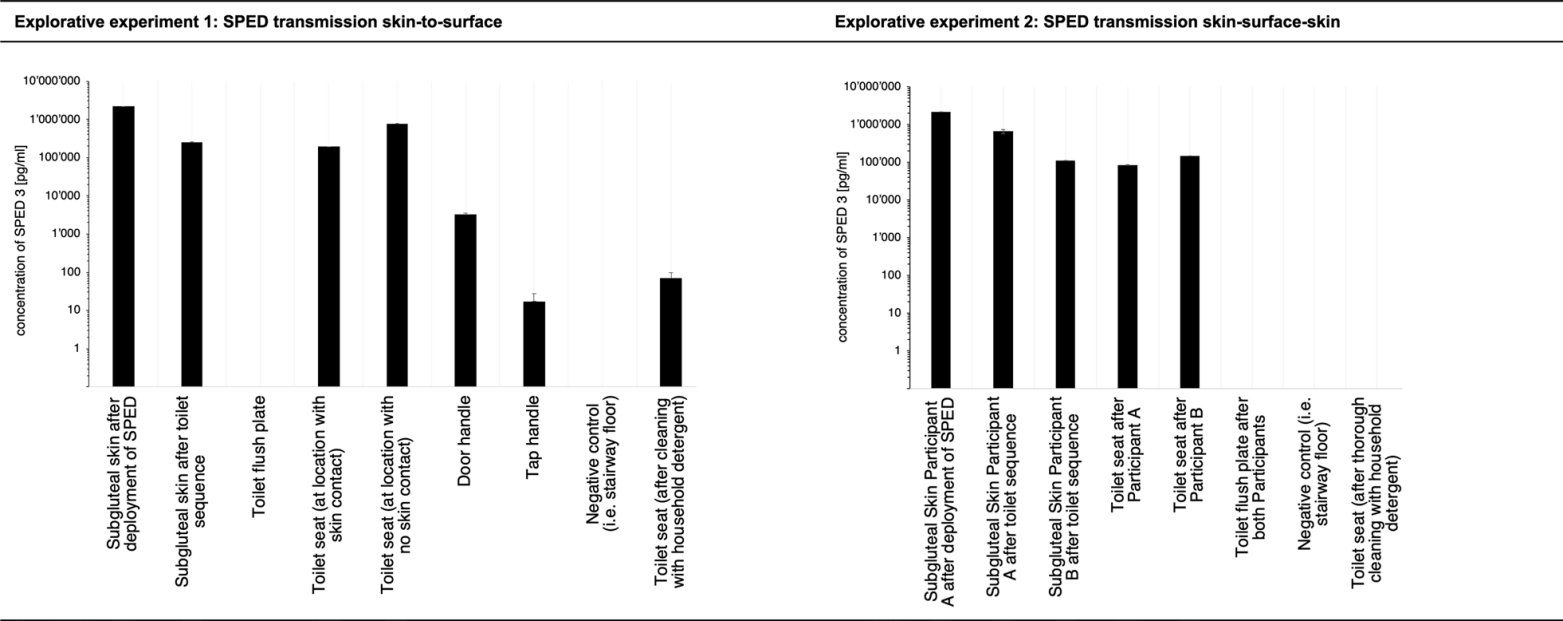
Results of the ‘Explorative experiments’. Explorative experiment 1: Transmission of SPED deployed to subgluteal human skin to inanimate objects such as the toilet seat or the door handle through a standardized toilet sequence; Explorative experiment 2: Transmission of SPED deployed on Participant-A’s subgluteal skin to Participant-B’s subgluteal region through successive use of the same toilet. The concentrations of SPED as measured by qPCR are displayed on a logarithmic scale. The baseline corresponds to the background concentration, meaning any positive value in the diagram corresponds to a signal above the baseline samples taken before SPED deployment. *SPED* silica nanoparticles with encapsulated DNA

Both explorative experiments successfully established transmission signals with the applied SPED amount and concentration from human skin to inanimate surfaces and back to skin (Fig. 3).

After testing different cleaning processes, we found a thorough cleaning process using water and a household detergent (Oekoplan, Coop) with multiple applications (≥ 5 times) each time using a new paper tissue eliminating SPED (Fig. 3). During all cleaning procedures gloves were worn to avoid recontamination. Microfiber cloths were not suitable to eliminate SPED.

### Patient experiment

**Fig. 4:**
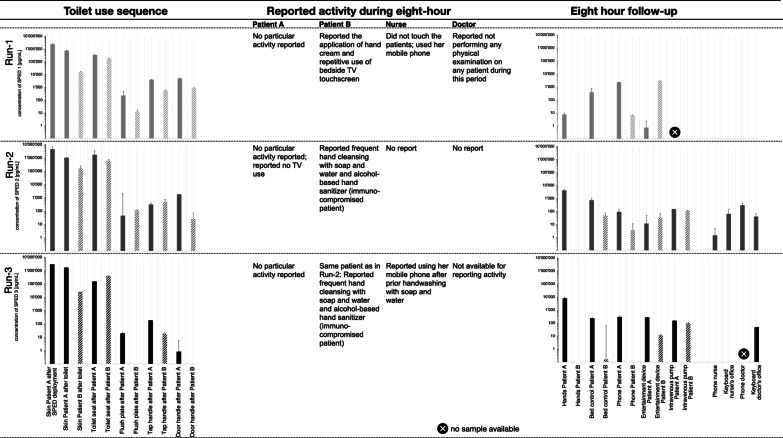
Results of the ‘Patient experiment’ (Run-1 to Run-3). SPED, silica nanoparticles with encapsulated DNA. Quantitative results of patient experiment involving a real-world toilet-use sequence. The concentrations of SPED as measured by qPCR from individual swab sites are displayed on a logarithmic scale. The baseline corresponds to the background concentration, meaning any positive value in the diagram corresponds to a signal above the baseline samples taken before SPED deployment

Overall, 133 swabs were collected during Run-1 to Run-3. The highest concentrations for 60 negative control samples before SPED deployment for Run-1, Run-2, and Run-3 were 3.01 × 10^–08^ mg/mL (SPED 3), 9.55 × 10^–09^ mg/mL (SPED 2) and 8.21 × 10^–09^ mg/mL (SPED-1), respectively, serving as run-specific positivity thresholds. Of the remaining 73 samples, 59 (80.8%) were positive (Fig. 4). Although the recovered SPED followed the logic of reduced concentrations with multiple touching sequences, there were considerable quantitative differences between the runs. Over all three runs, positivity rates were 100% on subgluteal skin, toilet seats, tap handles, and entertainment device controls, Patient-A’s hands; 83.3% on patient phones and bed controls; 80% on intravenous pumps; 75% on toilet flush plate and door handle, 66.6% on doctor’s keyboards; and 33.3%, on nurses’ or doctors’ phones, nurse’s keyboards, and 0% on Patient-Bs’ hands.

### Mobile device experiment

**Fig. 5:**
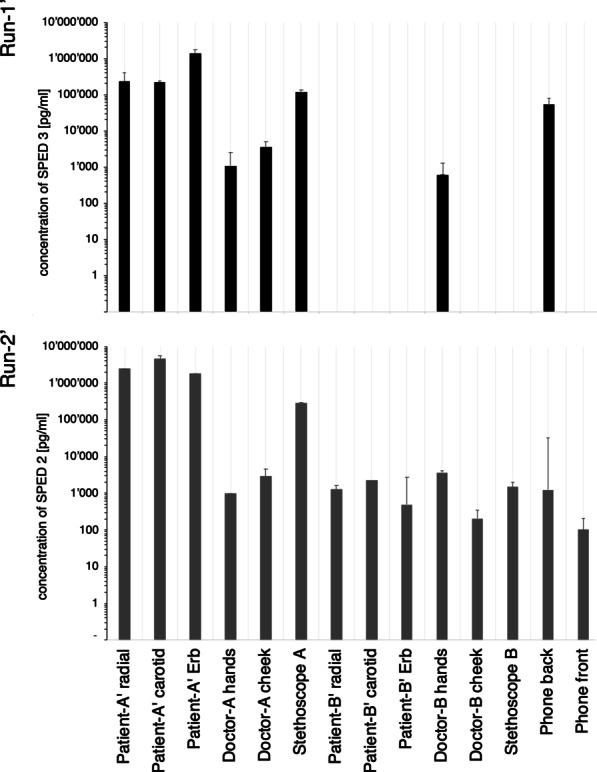
Results of the ‘Mobile device experiment’. Quantitative results of experiment mimicking a mobile device use scenario. The concentrations of SPED as measured by qPCR from individual swab sites are displayed on a logarithmic scale. The baseline corresponds to the background concentration, meaning any positive value in the diagram corresponds to a signal above the baseline samples taken before SPED deployment

**Fig. 6:**
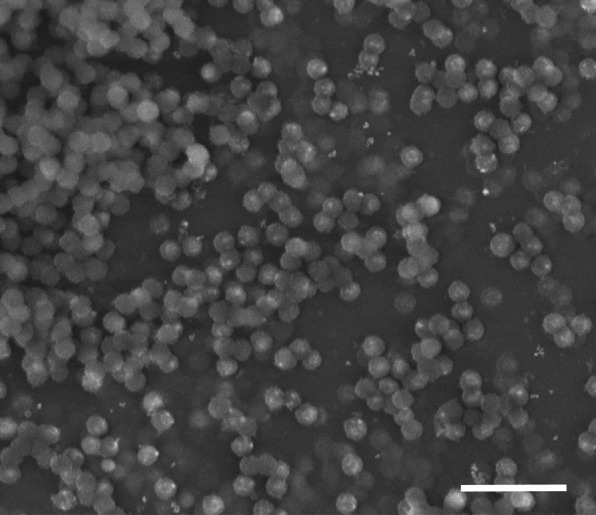
Scanning electron microscopy image of dried SPED. Scale bar indicates 500 nm

Overall, 56 samples were collected. Positivity thresholds in 28 baseline samples were 7.36 × 10^–7^ mg/mL (SPED-3) and 1.63E × 10^–7^ mg/mL (SPED-2) for Run-1′ and Run-2′, respectively. The baseline sample of Doctor-A’s hands in Run-1′ was accidently contaminated and ignored. Of the 22 post-experiment samples, not including the initial SPED deposition sites on Patient A′, 16 (72.7%) tested positive for SPED (Fig. 5).

## Discussion

After Scotoni et al. [25] explored SPED transmission in the laboratory and found their transmission behaviour to be similar to that of bacteria, the current study employed SPED for the first time in a real-life hospital environment. After establishing the method of skin contamination and sampling, we found SPED transmission from one patient to another via toilet use and spread to patient surroundings as far as distant HCWs’ mobile phones and keyboards consistently over all runs. An additional off-site experiment confirmed that HCW mobile phones indeed transmitted SPED between patients.

The patient experiment indicated SPED spreading from Patient-A’s subgluteal skin region to the toilet seat and from there to the subgluteal region of the roommate Patient-B. In addition, Patient-A contaminated the toilet flush plate, tap handle, and door handle with their hands with SPED which were acquired from a contaminated body part or environmental site beforehand. Patient-B became contaminated on the subgluteal skin site by exposure to the toilet seat. Of note, SPED were detected on the door handle in most of the cases, even though touching of the door handle occurred after handwashing in the study scenario. This implies that SPED had not been completely removed by handwashing. This contrasts the findings of Scotoni et al. [25] who investigated handwashing under laboratory conditions, but it positively simulates insufficient handwashing after toilet usage that has frequently been reported as typical population behaviour [43].

Eight hours after initial exposure, SPED have found their way to many surfaces in the immediate surroundings of the two patients and, in some instances reached the mobile phones and keyboards of the HCWs in charge of the patients, far from their room. Although SPED were always detectable on Patient-As’ hands, swabs of Patient-Bs’ hands were consistently negative. Nevertheless, SPED were found on all entertainment devices swabbed during the experiment, indicating a relevant but transient contamination of the patient’s hands. Swabs of Patient-A’s bed position controls, phones, entertainment devices, and intravenous pumps were uniformly positive, as were most swabs of Patient-Bs’ bed position controls, phones, and intravenous pumps. Patient-B tended to show lower SPED levels than Patient-A, consistent with the logic of a dilution effect over propagated spreading along a multi-touch transmission pathway (Fig. 4). The lower SPED concentration found on flush plates could be explained by an uneven distribution of SPED over the hands and the way toilet handles were touched, as shown by Scotoni et al. before [25].

Intravenous pumps, which are typically only operated by nurses, showed a positive signal for SPED in 80%. This echoes the findings by Huslage et al. [42], who considered these devices as “high-touch” surfaces and implied HCW hands as transmission hubs. Alternatively, but less likely, patients touched their intravenous pump themselves.

Pathogens found on toilet seats are often of faecal or skin origin including *Escherichia coli*, vancomycine-susceptible enterococci, VRE, and methicillin-resistant *Staphylococcus aureus* (MRSA) [44, 45]. Toilet seats have been suspected as culprits for VRE transmission [23] and individually attributed toilets have been associated with the control of VRE outbreaks [46, 47]. Transmission via toilet seats was, however, never formally investigated. Our results strongly support the presumable transmission of pathogens through a shared toilet seat.

Because we found a relevant concentration of SPED on a doctor’s mobile phone far from the study patients’ room and because mobile devices are increasingly used at the bedside, we decided to design a quasi-realistic patient care scenario that guaranteed SPED could only reach the second patient through the mobile phone. The different contamination levels found on the phone’s screen and back, again indicate the translation of hand touching into SPED detection patterns. Of note, the well-known risk of pathogen transmission through stethoscopes was echoed in this experiment by a higher SPED load. Thakur et al. [27] equally found stethoscopes to transmit a surrogate marker and MRSA. Thus, we agree with other authors who have suggested targeted cleaning of stethoscopes [48, 49] and phones [50–52] before.

This study has limitations. First, the number of conducted experiments did not allow to perform comparative statistics. Our goal was to use SPED for the first time in a real-life hospital setting and outbreak situation and to test if toilet seats need indeed to be considered in the spread of VRE or other potentially multi-drug resistant enteric flora. Second, as is the case with other surrogate tracers [27], habitual disinfection products do not inactivate SPED, while thorough cleaning with detergent and water does. This precludes mimicking real-life transmission perfectly, but instead allows to trace potential transmission pathways in the absence of disinfection procedures or, as our patient experiment shows, with the flawed execution of cleaning and disinfection procedures as it is often observed in real life. Further development of the SPED technology rendering the particles sensitive to disinfection agents might be feasible. Third, the extent of the spread of SPED ultimately depends on the quantity initially applied, but as Otter et al. [12] stated, the presence of pathogens at any concentration carries a risk for a transmission. Furthermore, the interpretation of relative quantity of recovered SPED must be taken with caution, since it depends on many factors including the swabbing technique. This does, however, not interfere with the discovery of transmission pathways. Fourth, by which intermediate transmission steps SPED reach the detection sites (e.g., the doctor’s keyboard or mobile phone) remains unknown. Therefore, time intervals and swabbing sites must be determined based on an initial hypothesis. Resolution can be increased by increasing the number of swabbing sites and rates as did Oelberg et al. [26], or by adding targeted evaluations as in our mobile device experiment. Fifth, being aware of an ongoing transmission experiment on their ward, HCW may have enhanced their infection prevention behaviour, e.g., use of alcohol-based handrub. The effect on spread of SPED would have been limited since SPED are not sensitive to alcohol. Moreover, this study was conducted during the SARS-CoV-2-pandemic, which has been associated with altered infection prevention behaviour [54, 55]. Sixth, as the name suggests, surrogate markers remain a substitute and will never behave exactly equal to microorganisms.

Transmission of microorganisms is not in itself negative, as it could play an important role in establishing and maintaining protective microbiota [53]. SPED could eventually play a role to gather more corresponding insights.

## Conclusion

In conclusion, SPED spread between patients through shared toilet use in a two-bed patient room, starting from a small, contaminated skin area in one patient. This finding highlighted the need for a reliable cleaning protocol, specifically as a potentially successful control element of an ongoing VRE outbreak. Intravenous pumps, mobile phones, and stethoscopes equally qualify as transmission hubs. And finally, the well-established transmission risk associated with patients’ and HCWs’ hands was confirmed. With this study, SPED were successfully applied in a real-life healthcare environment for the first time. As an immediate reaction to the results of this study we increased VRE outbreak control measures for common toilets and introduced wireless buzzers to alert cleaning staff of their use. Future development of the SPED tracer system could attempt to render SPED sensitive to common disinfection procedures and combine them with automated registration of human activity to increase natural fidelity.

## Data Availability

The datasets analysed during the current study are available from the corresponding author on reasonable request.

## Appendix

### Technical appendix

#### DNA barcode sequences

DNA barcodes were ordered as single strands at a length of 65 nucleotides, with 40% GC-content, as listed in Table 3. The sequences were synthesized by Microsynth AG (Balgach, Switzerland) and shipped in dried state. Previous to annealing, the single strands were dissolved in annealing buffer (Tris 10 mmol/L pH 7.5–8.0, EDTA 1 mmol/L, NaCl 50 mmol/L) to a final concentration of 5 g/L. For annealing, the single strands were mixed together in equal parts, treated at 95 °C for 5 min and slowly cooled down to room temperature.

**Table 3:** DNA sequences used for SPED synthesis

Name	Sequence
SPED-1 forward	TTATGGGCTCTAAGGATCTCTTCGTTGTCGTTAGGTTCCTGCGTTTTTCGATTCGAGGGTGAGTT
SPED-1 reverse	AACTCACCCTCGAATCGAAAAACGCAGGAACCTAACGACAACGAAGAGATCCTTAGAGCCCATAA
SPED-2 forward	TATGCGCCTTTATACTCTTATAGGTATCCTGTTGCTGGCACTTTTTTCTAGCAAAGTCTTCTCCT
SPED-2 reverse	AGGAGAAGACTTTGCTAGAAAAAAGTGCCAGCAACAGGATACCTATAAGAGTATAAAGGCGCATA
SPED-3 forward	TAGCTCGTTCATAGAATCACTTCGCCGTACTCAACGTAGTGGTTTTTGTTTAGCTCAAACAGGTT
SPED-3 reverse	AACCTGTTTGAGCTAAACAAAAACCACTACGTTGAGTACGGCGAAGTGATTCTATGAACGAGCTA

#### SPED synthesis

Per batch of DNA encapsulation, 4 × 4 mL of silica nanoparticles (110 nm, 50 mg/mL in isopropanol; Pinfire, Frankfurt a. M., Germany) were surface-functionalized in 4 falcon tubes. For functionalization, 40 µg of *N*-trimethoxysilylpropyl-*N*,*N*,*N*-trimethylammonium chloride (TMAPS) (50 wt% in methanol; abcr, Karlsruhe, Germany) were added, followed by 12 h of shaking at room temperature, at 900 rotations per minute (rpm). Next, a 2 mL batch of 150 ng/µL of corresponding annealed DNA (sequences see Table 3) was added to 200 mL ultrapure water (mQ; type 1, 18.2 MΩ·cm at 24 °C, Milli-Q®; Merck, Darmstadt, Germany), followed by adding 0.4 g of the functionalized particles and vortexing for 10 s. Subsequently, 4 µL TMAPS were added, before vortexing and sonicating for 20 s. Then, 62.5 µL of tetraethyl orthosilicate (TEOS) (≥ 99.0%; Sigma-Aldrich, St. Louis, Missouri, USA) were added. The mixture was shaken for 5 h at 600 rpm. In a further step, 10 mL iPrOH and 5.9 mL TEOS were mixed with 484.1 mL ultrapure water and combined with the previous suspension. The batch was then stirred for 4 days at 600 rpm. Three different batches were synthesized, each labeled with its own DNA barcode.

#### SPED characterization

DNA loading of the SPED batches was estimated by measurement of DNA concentration in solution before and after the reaction on a photometer (NanoDrop 2000c; Thermo Fisher Scientific, Waltham, Massachusetts, US), the difference being the amount of loaded DNA. Hydrodynamic size distributions of SPED in suspension (~ 2 mg/mL in mQ water) were measured using a dispersion analyser (LUMiSizer, 470 nm light source; LUM GmbH, Berlin, Germany). Transmission profiles were recorded in time intervals of 5 s, at a rotational speed of 3000–4000 rpm, for 2 h. Statistical data analysis was performed by SEPView® software provided with the dispersion analyser. For scanning electron microscopy (SEM), SPED were prepared at a concentration of 0.1 mg/mL in iPrOH, loaded on a STEM C-grid and dried under infrared light. Imaging was performed on a NovaNanoSEM450 device (Field Electron and Ion Company, Hillsboro, Oregon, US) at a voltage of 20 kV.

#### Recovery and processing of SPED

In our experiments SPED were diluted in ultrapure MilliQ water (type 1, 18.2 MΩ·cm at 24 °C, Milli-Q®; Merck, Darmstadt, Germany) to a working concentration of 1 mg/mL, of which 3 mL were used for patient colonization. To detect SPED on skin or surfaces, a similar swabbing technique as established by Scotoni et al. [25] was applied. The swabbing technique included cotton swabs (Naturaline Wattestäbchen, Steinfels Swiss, Winterthur, Switzerland) premoistened in 20% glycerol solution (99+% Glycerol 1 L, VWR chemicals bvba, Leuven, Belgium; 20 vol% in mQ water) that were rolled three times over a pre-defined area and then added to 2 mL microcentrifugation tubes (Eppendorf AG, Hamburg, Germany) pre-filled with 200 µL mQ water and stored at room temperature. To fit the cotton swabs in the tubes, swabs were shortened with scissors. All samples were transported to the laboratory for further processing.

#### Quantification of SPED

The swabs obtained at the experimental site were stored at room temperature for a maximal duration of 14 h before being processed. For subsequent SPED quantification, each sample was ultrasonicated for 15 min and briefly spun down in a minicentrifuge to clear the SPED from the cotton swab as much as possible. For the etching process 2 vol% of buffered oxide etch, consisting of 0.03 wt% ammonium hydrogen difluoride (NH_4_FHF, pure; Merck, Darmstadt, USA) and 0.02 wt% ammonium fluoride (NH_4_F, puriss.; Sigma-Aldrich, St. Louis, Missouri, USA), was added to the sample liquid to release the DNA from the silica particles. All samples were ultrasonicated again for 15 min after adding BOE.

The suspension was then analyzed by real-time quantitative PCR (LightCycler® 96, Roche Molecular Systems, Pleasanton, USA) with SYBR® Green Master Mix (KAPA SYBR FAST qPCR master mix universal (2x, Kapa Biosystems, cat. no. KK4601)). The qPCR total reaction volume was 20 µL made up of 5 µL sample solution, 1 µL each of 10 µM forward and reverse primer stock solutions (Microysynth AG, Balgach, Switzerland, Table 4),

**Table 4:** Primer (forward and reverse) for each batch of SPED for qPCR

GM-06-S1 (SPED-1)	Primer forward ATGGGCTCTAAGGATCTC Primer reverse CTCACCCTCGAATCGAA
GM-06-S2 (SPED-2)	Primer forward ATGCGCCTTTATACTCTTA Primer reverse GGAGAAGACTTTGCTAGAA
GM-06-S3 (SPED-3)	Primer forward AGCTCGTTCATAGAATCAC Primer reverse ACCTGTTTGAGCTAAACAA

10 µL mastermix, and 3 µL mQ water. The qPCR program contained a preincubation for 240 s at 95 °C, followed by 40 cycles of a 3-step amplification protocol (2 s at 95 °C, 12 s at 60 °C, 4 s at 72 °C). Technical triplicates were measured for each sample, using no-template controls as PCR negatives. Further calculations were based on qPCR cycle values (Cq value) provided by LightCycler96 Software standard protocol.

#### Calculation of SPED concentration per sample

To quantify the amount of SPED in each sample, calibration curves were measured. For each batch of SPED used in our experiments a dilution series with 5 to 6 different concentrations (10^–3^, 10^–4^ mg/mL, etc.) was measured to assign established particle concentrations to the according cycle value from the qPCR.

The qPCR cycle values of the dilution series were plotted against the logarithmic particle concentration (in mg/mL resulting in a calibration curve. Use of linear regression of the single log chart generates a curve in the form of *y* = *ax* + *b* (y as the cycle value and x as the ln of the concentration), consequently the concentration equals e^y/m−b^.

The concentration of a given sample was calculated using the above formula with the respective regression parameters (Table 5). Error bars are based on the standard deviation of technical triplicates in qPCR data.

**Table 5:** Regression parameters for calculation of SPED concentration with the formula: y = ax + b (y as the cycle value and x as the ln of the concentration)

Batch	a	b	R^2^ (coefficient of determination)
SPED-1 (GM-06-S1)	− 1.404	− 4.429	0.994
SPED-2 (GM-06-S2)	− 1.645	− 5.397	0.999
SPED-3 (GM-06-S3)	− 1.852	− 6.345	0.999

#### Evaluation of SPED and threshold

Prior to each experiment every sampling site was swabbed as a blank sample. The blank sample with the lowest qPCR cycle value and thus, the highest load of SPED was used as threshold for the according experiment. The cut-off value was calculated based on the qPCR cycle value plus the standard deviation of the technical triplicates. Sample concentration above threshold were considered as *positive* for SPED, values below as *negative*.

#### Scanning electron microscopy SEM

SPED were prepared as a suspension of 0.1 mg/mL in 2-propanol, loaded on a STEM C-grid and dried under infrared light. The samples were imaged on a NovaNanoSEM450 device (Field Electron and Ion Company, Hillsboro, Oregon, US) at a voltage of 20 kV at 52,480× magnification (Fig. 6).

## Abbreviations

aOR: Adjusted odds ratio
HCAI: Healthcare associated infections
HCW: Healthcare worker
HCWH: Healthcare worker’s hands
HH: Hand hygiene
mQ water: Ultrapure MilliQ water (type 1, 18.2 MΩ·cm at 24 °C, Milli-Q®; Merck, Darmstadt, Germany)
MRSA: Methicillin-resistant *Staphylococcus aureus*
qPCR: Quantitative polymerase-chain-reaction
SPED: Silica particles with encapsulated DNA
VRE: Vancomycin-resistant enterococci
